# Four-factor prothrombin complex concentrate (Beriplex® P/N) is superior to three-factor prothrombin complex concentrate for reversal of coumarin anticoagulation

**DOI:** 10.1186/cc14427

**Published:** 2015-03-16

**Authors:** E Herzog, F Kaspereit, W Krege, P Niebl, G Dickneite

**Affiliations:** 1 CSL Behring GmbH, Marburg, Germany

## Introduction

The study was conducted as a head-to-head comparison of a four-factor prothrombin complex concentrate (4F-PCC) and two different three-factor PCCs (3F-PCC) for effective reversal of vitamin K antagonist (VKA)-induced anticoagulation using an established rat model of acute bleeding [1]. The 4F-PCC (containing the human coagulation factors II, VII, IX and X) is indicated for the urgent reversal of acquired coagulation factor deficiency induced by VKA therapy in adult patients with acute major bleeding. In contrast, the 3F-PCCs (containing factors II, IX, × and only minimal VII) are indicated for the prevention and control of hemorrhagic episodes in hemophilia B patients. Nevertheless, the use of 3F-PCC for correcting hemostasis following warfarin overdose has been discussed but the lack of factor VII in these 3F-PCC products has raised questions about efficacy.

## Methods

Rats received an oral dose of 2.5 mg/kg phenprocoumon. At 15.75 hours post dosing, animals were treated with a single intravenous dose of saline, 4F-PCC (Beriplex® P/N, Kcentra®; CSL Behring) or 3F-PCC (Bebulin® VH; Baxter and Profilnine® SD; Grifols). Study endpoints included bleeding following tail clip, activated partial thromboplastin time (aPTT), and prothrombin time (PT). In addition, the plasma levels of vitamin K-dependent coagulation factors were determined.

## Results

Acute coumarin anticoagulation of rats induced a rise in median bleeding time by ≥2-fold from an average of 823 to 1,800 seconds (maximum observation period) compared with untreated animals. In parallel, PT and aPTT were prolonged from 8.9 to 29.9 seconds and from 14.5 to 25.5 seconds, respectively. Treatment with 4F-PCC was able to fully and statistically significantly reverse bleeding, achieving average bleeding times of 676 seconds. In parallel, the elevation in PT was reduced to 15.1 seconds. In contrast, the two 3F-PCC products were not or only partially able to reduce coumarin-induced bleeding with average bleeding times of 1,398 and 1,708 seconds post treatment, respectively. This also correlated with inferior reductions in PT which achieved minimum levels of 23.8 and 29.5 seconds, respectively. There was no reduction in aPTT seen for any treatment option.

## Conclusion

In conclusion, this first direct comparison of 4F-PCC and 3F-PCCs for the reversal of VKA anticoagulation in a rat model of acute bleeding suggests that replenishment of all four vitamin K-dependent coagulation factors including factor VII as achieved using a 4F-PCC may result in superior efficacy compared with the use of 3F-PCCs.
